# Genome-wide profiling of DNA 5-hydroxymethylcytosine during rat Sertoli cell maturation

**DOI:** 10.1038/celldisc.2017.13

**Published:** 2017-05-09

**Authors:** Miriam Landfors, Jostein Johansen, Jan Magnus Aronsen, Cathrine Broberg Vågbø, Louis C Doré, Chuan He, Ivar Sjaastad, Pål Sætrom, Péter Fedorcsák, John Arne Dahl, Håvard Aanes, Markus Fußer, Arne Klungland

**Affiliations:** 1 Department of Microbiology, Institute of Medical Microbiology, Rikshospitalet, Oslo University Hospital, Oslo, Norway; 2 Department of Cancer Research and Molecular Medicine, NTNU—Norwegian University of Science and Technology, Trondheim, Norway; 3 Institute for Experimental Medical Research, Oslo University Hospital and University of Oslo, Oslo, Norway; 4 Bjørknes College, Oslo, Norway; 5 Department of Chemistry, Institute for Biophysical Dynamics, Howard Hughes Medical Institute, The University of Chicago, Chicago, IL, USA; 6 Department of Computer and Information Science, NTNU—Norwegian University of Science and Technology, Trondheim, Norway; 7 Department of Reproductive Medicine, Oslo University Hospital, Oslo, Norway; 8 Department of Tumor Biology, Institute for Cancer Research, Oslo University Hospital, Oslo, Norway; 9 Department of Molecular Medicine, Institute of Basic Medical Sciences, University of Oslo, Oslo, Norway

**Keywords:** 5-hydroxymethylcytosine, epigenetics, DNA modification, Sertoli cells, cellular maturation

## Abstract

Sertoli cells have dual roles during the cells’ lifetime. In the juvenile mammal, Sertoli cells proliferate and create the structure of the testis, and during puberty they cease to proliferate and take on the adult role of supporting germ cells through spermatogenesis. Accordingly, many genes expressed in Sertoli cells during testis formation are repressed during spermatogenesis. 5-Hydroxymethylcytosine (5hmC) is a DNA modification enzymatically generated from 5mC and present in all investigated mammalian tissues at varying levels. Using mass spectrometry and immunofluorescence staining we identified a substantial Sertoli cell-specific global 5hmC increase during rat puberty. Chemical labeling, pull-down and sequencing of 5hmC-containing genomic DNA from juvenile and adult rat Sertoli cells revealed that genes that lose or gain 5hmC belong to different functional pathways and mirror the functions of the cells in the two different states. Loss of 5hmC is associated with genes involved in development and cell structure, whereas gain of 5hmC is associated with genes involved in cellular pathways pertaining to the function of the adult Sertoli cells. This redistribution during maturation shows that 5hmC is a dynamic nucleotide modification, correlated to gene expression.

## Introduction

There are ∼200 different cell types in the body of a mammal. Cellular identity is linked to and maintained by a unique epigenetic make-up. During differentiation, cells acquire epigenetic barriers that prevent cells from transitioning into a less or more differentiated state [1].

5-hydroxymethylcytosine (5hmC) was identified in 2009 as a frequent modification of mammalian genomic DNA, and was reported to be particularly enriched in the brain [2]. The ten–eleven translocation (TET) enzymes, belonging to the Fe(II) and 2-oxoglutarate-dependent TET family, were found to oxidize 5mC to 5hmC in mouse [3, 4]. 5hmC is present in all mammalian tissues investigated to date [5], and its global levels differ significantly among tissues and cell types [6]. Indeed, the tissue type is the main determinant for the 5hmC level of any given gene [6]. Cell states also seem to exhibit differences in 5hmC levels: in embryonic stem cells, global 5hmC content decreases during differentiation [7], whereas tissue-specific neuronal cells acquire 5hmC upon differentiation [8]. Furthermore, conserved features of the 5hmC distribution in the genome have emerged: 5hmC is enriched in gene bodies and depleted in intergenic regions [8, 9]. In addition to its role as epigenetic mark, it has been suggested that the oxidation of 5mC to 5hmC is the first step in a consecutive demethylation pathway that ultimately reintroduces a cytosine. As a part of this demethylation pathway, TET enzymes convert 5hmC further to 5-formylcytosine and 5-carboxylcytosine, which would serve as substrates for DNA glycosylases of the base excision repair pathway that restores cytosine [10–12]. However, the distribution of 5hmC has been found to be unique and not simply a consequence of 5mC distribution [13], with 5hmC being more selectively enriched in genes than 5mC [14]. In addition, while 5hmC levels vary strongly among different cell types, levels of 5mC are relatively similar across tissues [5].

Sertoli cells have an important role in the formation and development of the mammalian testis, being the first cells to differentiate in the fetal gonad [15, 16]. The formation of the basic structures of the testis is followed by rapid proliferation of Sertoli cells [15]. At the time of puberty, Sertoli cells mature, lose the ability to proliferate and take on the role of supporting the developing germ cells with nutrients and regulatory factors [16]. The supporting functions of adult Sertoli cells are largely absent during the prepubertal period [17]; consequently, many of the genes expressed in Sertoli cells during the period of testis formation are not expressed during later spermatogenesis and *vice versa* [18].

The dual role of Sertoli cells, that is, formation of the testis before puberty and support of spermatogenesis during and after puberty, and the earlier finding that replicative and post-replicative murine cells exhibit different levels of 5hmC [5], prompted us to study the 5hmC distribution in these two cell states. We wanted to investigate whether maturation of Sertoli cells affects global 5hmC content. Moreover, we sought to explore the difference in genomic distribution of 5hmC before and after maturation and identify genes characterized by either gain or loss of 5hmC.

## Results

### Isolation of Sertoli cells

**Figure 1:**
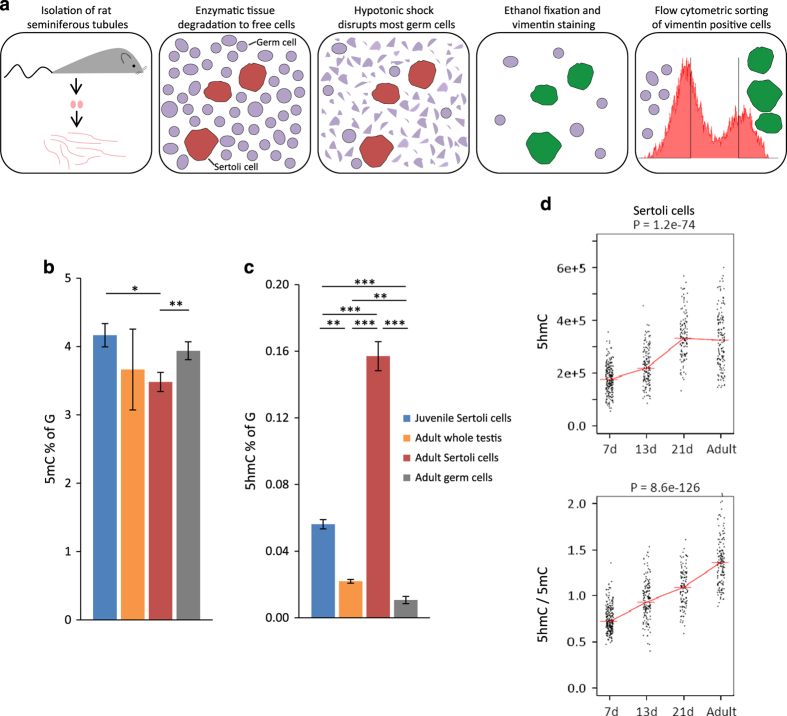
Isolation of adult Sertoli cells and quantification of global levels of 5mC and 5hmC. (**a**) Schematic overview of the isolation of adult Sertoli cells from whole testis. Quantification of global levels of (**b**) 5mC and (**c**) 5hmC in Sertoli cells. Mass spectrometric quantification of modified cytosines as percentage of guanine. Student’s *t*-test, ***P-value≤0.0001; **P-value≤0.005; *P-value≤0.01; error bars represent s.d.; *n*=3. (**d**) Immunofluorescence quantification of 5hmC and 5hmC/5mC, respectively, at ages 7 days, 13 days, 21 days and 15 weeks (adult). Signal intensity measured relative to DAPI intensity (arbitrary units), see Supplementary Figure S2 for details; *n*=250–300 per time point.

In order to ascertain that the density and distribution of 5hmC reflect the physiological state in the organism, we used primary isolates of Sertoli cells without extended culture or proliferation *in vitro*. Adult Sertoli cells were isolated using enzymatic tissue digestion and hypotonic shock, followed by fluorescence-activated cell sorting (Figure 1a and Supplementary Figure S1). The cells were labeled for vimentin, which in the context of the seminiferous tubule is a marker for Sertoli cells (Supplementary Figure S1B), and sorted on the basis of signal intensity (Supplementary Figure S1C). In order to estimate cell purity, the sorted cells were labeled with SOX9, an additional Sertoli cell-specific marker, which localized to the vimentin-stained cells (Supplementary Figure S1E). The purity of the sorted Sertoli cell fraction was consistently ∼95% (Supplementary Figure S1D).

### The global level of 5hmC increases during rat Sertoli cell maturation

Using liquid chromatography-tandem mass spectrometry (LC-MS/MS) we determined the global levels of genomic 5mC and 5hmC in rat Sertoli cells from juvenile (7 days) and adult animals (Figure 1b and c). The amount of 5mC was lower in adult than in juvenile Sertoli cells, 3.5% 5mC of G compared to 4.2% 5mC of G, a decrease of ∼16.4%. In the adult animals, germ cells exhibited a 13.1% higher level of 5mC than Sertoli cells (Figure 1b). As for 5hmC, DNA derived from adult Sertoli cells contained considerably more 5hmC than DNA from juvenile Sertoli cells (Figure 1c). We observed a 2.8-fold increase in 5hmC density during puberty in Sertoli cells, from 0.056 to 0.157% 5hmC of G.

The level of 5hmC in adult Sertoli cells (0.157% 5hmC of G) exceeded the level of the whole testis (0.022% 5hmC of G) by ∼7.2 times and the level of adult germ cells (0.011% 5hmC of G) by ∼14.7 times. The level of 5hmC in adult germ cells was only 48.7% of that of the level of the adult whole testis. Sertoli cells make up a very small proportion of the cells in the seminiferous tubule of adult testis. Quantitative data from mouse show that Sertoli cells represent ∼2% of the cells in the adult seminiferous tubule [19]. The higher 5hmC level in whole testis as compared to germ cells alone is likely due to the contribution from Sertoli cells.

### 5hmC increase confirmed using immunofluorescence

The increase in 5hmC density during puberty was further confirmed by immunofluorescence quantification (Figure 1d). The 5hmC-specific fluorescence increased in Sertoli cells from day 7 to day 21, and remained unchanged from 21 days until adulthood; this pattern differed from the pattern observed in other testicular cell types, including peritubular cells and spermatogonia, which did not display increased 5hmC-specific fluorescence (Supplementary Figure S2 displays all cell types analyzed). In addition, while the levels of 5hmC in Sertoli cells remained comparable from 21 days to adulthood, peritubular cells, spermatogonia and the emerging spermatocytes displayed reduced 5hmC levels between these time points. Notably, the variation of 5hmC levels in the Sertoli cells was increased in adult compared to juvenile animals.

### Mapping of 5hmC in the juvenile and adult Sertoli cell genome

**Figure 2:**
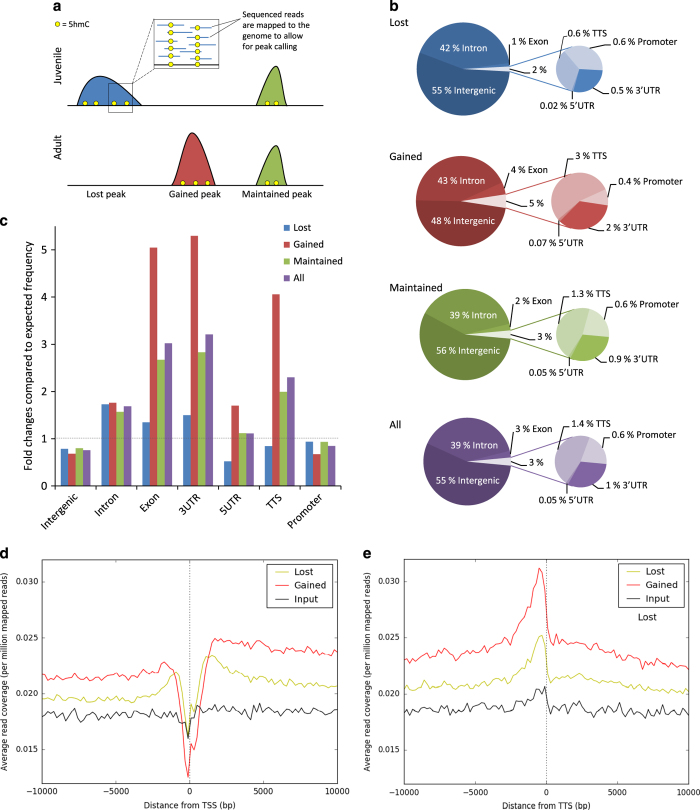
Mapping the sequenced reads to the rat genome. (**a**) Schematic overview, sequenced reads are mapped to the genome to allow for peak calling; peaks are lost, gained or maintained as juvenile Sertoli cells mature into adulthood. (**b**) Representation of the peaks in the gene regions. (**c**) Fold changes compared to the expected frequency of each gene region in the rat genome; expected value is 1. (**d**) Average read coverage around the TSS and (**e**) transcription termination site (TTS). The input sample was generated by pooling equal amounts from each of the six input samples.

Genomic DNA was isolated from juvenile and adult rat Sertoli cells. The DNA containing 5hmC was chemically labeled, pulled down and sequenced as described in Materials and methods. The sequenced reads were then mapped to the rat genome and peaks were called using *SICER*, see Figure 2a for a schematic representation. There were 84 890 and 56 074 peaks in the adult and 7d samples, respectively, which resulted in a total of 98 208 identified peaks in the combined data. Of these, 43 870 peaks were intragenic and localized to 10 725 genes (Table 1). The 5hmC peaks were classified into three groups: peaks that were lost during maturation (9 465); peaks that were gained during maturation (10 116); and peaks that were maintained and appeared in both age groups (78 627). The number of peaks that mapped to intragenic regions and were lost was 4 258; the number of intragenic peaks that were gained was 5 275; and there were 34 337 intragenic peaks that were maintained during maturation. The numbers of input and filtered reads are shown in Supplementary Table S1. These counts are in agreement with data from previous studies using this 5hmC sequencing technology [20].

### Gene region enrichment differs among peak groups

The three peak groups we distinguished, that is peaks lost, gained and maintained during maturation, differed in the proportion of peaks that mapped to different gene regions. The gained peaks, which were only present in the adult samples, were more enriched in gene regions other than intronic and intergenic (Figure 2b). Figure 2c shows the fold changes compared to the expected frequency of each gene region in the genome. The density of 5hmC in intergenic regions was lower than expected in all peak groups. The differences among the groups were small in the intergenic and intronic regions. All, but particularly the gained, 5hmC peaks were under-represented at promoter regions. In exons, 3′ untranslated regions (UTR), 5′UTR and transcription termination sites, the gained 5hmC peaks exhibited a markedly higher fold change from expected than the maintained and the lost 5hmC peaks. For this latter group, 5hmC is under-represented in the category 5′UTR. The average read coverage in the vicinity of the transcription start site (TSS) and the transcription termination site is shown in Figure 2d and e. We observed a drop in 5hmC levels at the TSS and an increase in 5hmC levels immediately upstream of the transcription termination site for both lost and gained peaks.

### Peak length and peak count vary among peak groups

The length of the 5hmC peaks differed among the groups. The peaks that were lost during maturation were significantly longer than both the peaks that were gained and the peaks that were maintained (Supplementary Figure S3A). The number of peaks per gene also differed among the groups (Supplementary Figure S3B). Genes that lost 5hmC compared to genes that gained 5hmC contained on average more peaks; the genes with maintained peaks had more peaks on average than genes in the other groups. Supplementary Figure S3C shows the mean of all log2-count per million (log2CPM) peak intensities per chromosome for the lost, maintained and gained groups. For all chromosomes, except chromosome 12, the lost group shows higher intensity than the gained and maintained groups. Chromosome Y’s lost group (20 peaks) shows much higher intensity on average compared to the other chromosomes. Supplementary Figure S3D and E indicates the percentage of gained and lost 5mC and 5hmC peaks per chromosome. The Y chromosome both loses and gains 5mC. However, it does not gain any 5hmC during maturation, but there is a substantial loss of 5hmC.

### Lost and gained 5hmC is enriched in genes belonging to different functional pathways in juvenile and adult Sertoli cells

**Figure 3:**
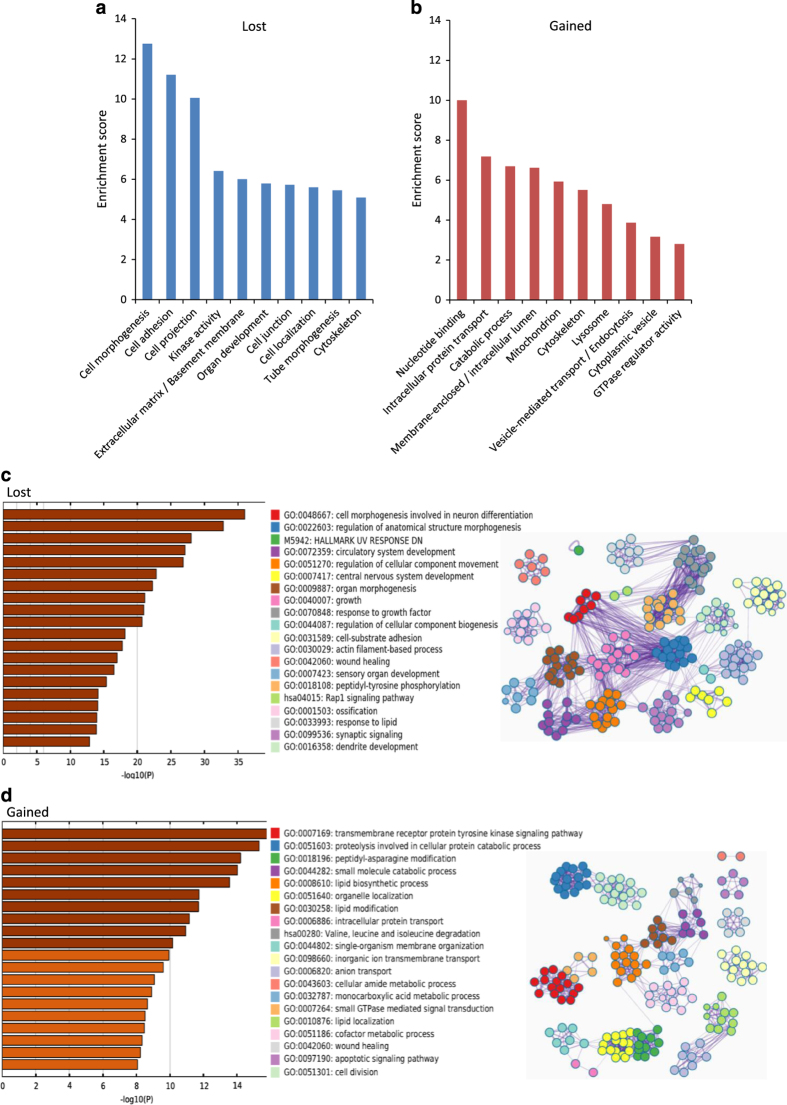
Visualization of the pathways enriched with 5hmC that is lost or gained during maturation of Sertoli cells. (**a**) DAVID and (**c**) Metascape gene ontology searches based on genes that contain 5hmC peaks that are lost. (**b**) DAVID and (**d**) Metascape gene ontology searches based on genes that contain 5hmC peaks that are gained. Metascape selects up to 10 terms within each of the 20 top-scoring clusters, and then connect all terms pairs with Kappa similarity above 0.3. Network visualized with Cytoscape; each node represents one enriched term; edges link similar terms. Only one term within each cluster is chosen to show its label. −log10(P) values higher than 10 are shown in brown bars; lower than 10 are shown in orange.

Gene ontology analyses using the DAVID database on genes with lost or gained 5hmC peaks revealed that these two peak groups were associated with genes involved in distinct cellular pathways (Figure 3).

Ten of twenty pathway clusters with the highest enrichment scores in the lost and gained 5hmC peak groups are shown in Figure 3a and b, and the complete set of pathways is listed in Supplementary Table S2. The 5hmC peaks that were lost during maturation mapped to genes associated with morphogenesis and cell structure (Figure 3a). Genes related to cell adhesion, cell projection, cytoskeleton and localization of the cell displayed high enrichment scores, as did genes related to tube morphogenesis. Corresponding searches on 5hmC peaks that were gained during Sertoli cell maturation identified pathways associated with transportation, catabolic processes, mitochondrion and endocytosis (Figure 3b).

To confirm these observations, we performed additional analyses using the Metascape database. The results corroborated data obtained using the DAVID database and confirmed a clear dichotomy between enriched pathways in the groups with gained or lost 5hmC peaks (Figure 3c and d). The pathways enriched in the lost 5hmC peak group included morphogenesis- and development-related terms, whereas the gained 5hmC peak group was associated with genes involved in metabolic processes and transport. We observed strong connections among cellular pathways related to genes with lost 5hmC peaks, as visualized by enrichment networks. Database searches for gained 5hmC peaks gave considerably lower significance scores than searches on lost 5hmC peaks, indicating a wider distribution of affected pathways.

In order to consider all 5hmC peaks present in the juvenile animals, we included maintained and lost peaks in a juvenile data set. A corresponding adult data set was created including maintained and gained peaks. These data sets were probed for enriched pathways using gprofileR (Supplementary Figure S4). We observed a substantial overlap in metabolism-related terms between the juvenile and adult data sets, and noted the absence of developmental terms in the adult group.

### 5hmC enrichment in genes that are upregulated in immature Sertoli cells

**Figure 4:**
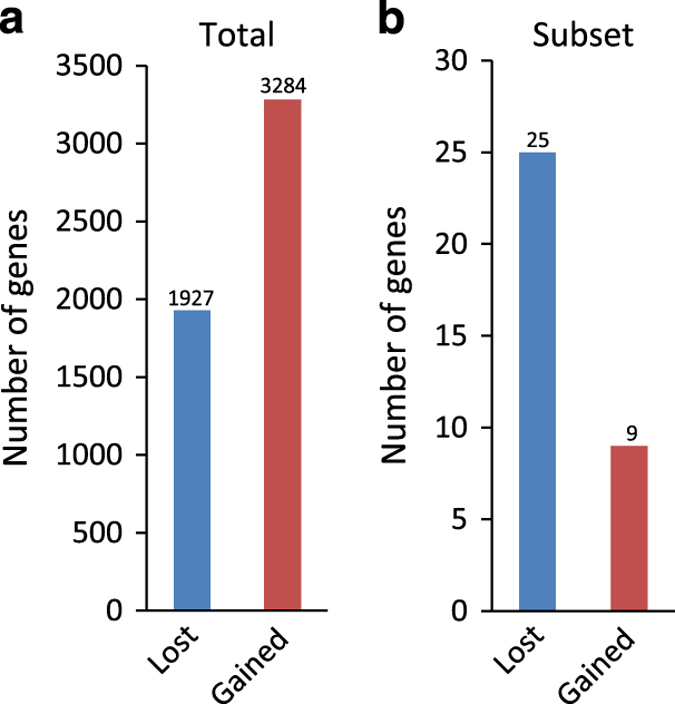
Number of genes that contain 5hmC that is lost and gained during maturation of Sertoli cells. (**a**) Total number of genes that lose and gain 5hmC, respectively. (**b**) Number of genes that lose and gain 5hmC in the subset of 101 genes that have been found to be highly expressed in immature Sertoli cells.

We further investigated the presence of 5hmC modification in genes known to be upregulated in immature Sertoli cells [21] (Supplementary Table S3). Of the 101 upregulated genes identified by Ryser *et al.* [21], 36 were pulled down for 5hmC in our experiments. Interestingly, 25 of these genes contained intragenic 5hmC peaks that were lost during Sertoli cell maturation. For the total pull-down, there are 70% more genes that contained gained 5hmC peaks than lost 5hmC peaks (see Figure 4a). However, for the subset of 101 genes identified as highly expressed in immature Sertoli cells, 64% fewer genes contained gained peaks than lost peaks (see Figure 4b). Hence, there is a significantly larger proportion of genes that lose 5hmC during maturation among the genes that are highly expressed in immature Sertoli cells than in the total pull-down sample (odds ratio of 4.8 for lost compared to gained; *P*=0.00002, Fisher’s exact test). The median number of peaks for this group of 25 highly expressed genes with at least one lost peak is 7 (mean 8). In contrast, the median number of peaks for the group of nine genes that contained gained peaks was 4 (mean 3). This reflects the previously mentioned pattern (Supplementary Figure S3): genes with lost peaks have more peaks than other genes.

## Discussion

The pubertal maturation of Sertoli cells is associated with cessation of proliferation, alterations in gene transcription and protein expression, construction of inter-Sertoli cell tight junctions, establishment of the blood–testis barrier and the capacity to sustain spermatogenesis [17, 22]. The functions of adult Sertoli cells that support spermatogenesis are largely absent during the prepubertal period [17]. The maturation of the Sertoli cell is considered to be a step-wise process and failure to undergo an early step may prevent or interfere with subsequent steps [17]. Erroneous maturation of Sertoli cells into the terminally differentiated adult state has been shown to be the cause of reduced fertility in men [17]. Indeed, functionally immature Sertoli cells are observed in men with infertility [23, 24].

It has been shown that the presence and state of the germ cells regulate the gene expression of the nurturing Sertoli cells [25, 26], and complex interactions between somatic Sertoli cells and germ cells are required for proper spermatogenesis [27]. Sertoli cells in conventional culture do not maintain these crucial Sertoli cell–germ cell interactions, nor do these cells polarize or express tight junctions that are essential for their proper function *in vivo* [16]. The absence of germ cells induces regression to immature traits in cultured Sertoli cells [17]. Furthermore, global 5hmC levels are reduced in cultured cells compared to uncultured primary samples, and 5hmC content is rapidly diminished with each passage [6]. In order to avoid spurious effects of cell culture *in vitro,* we studied primary isolates of Sertoli cells that were enriched with fluorescence-activated cell sorting.

### The global level of 5hmC increases during rat Sertoli cell maturation and gene regions differ in 5hmC enrichment

We determined the total amount of 5mC and 5hmC in rat testis, and found that the variability of 5mC levels was lower according to cell types than the variability of 5hmC levels. This pattern of stable 5mC distribution and tissue-dependent variation in 5hmC has been previously described [5, 6]. The level of 5mC is comparable to data obtained from mouse testis [5]. The level of 5hmC in adult whole-rat testis (0.022% 5hmC of G) is also in agreement with previous results obtained from adult mouse testis (~0.025% 5hmC of G) [5]. We observed a threefold increase of 5hmC levels in the adult compared to the immature Sertoli cells, which may be related to the post-mitotic state of the Sertoli cells. Indeed, the postnatal day 7 mouse cerebellum, which is characterized by massive cell proliferation, has low 5hmC levels compared to the adult, non-proliferating cerebellum [2, 8, 20].

The pattern of 5hmC levels shown in Figure 1d suggests that the 5hmC level remains stable beyond day 21 of life. At this time point Sertoli cells have lost proliferative ability [28], and formed the inter-Sertoli cell tight junctions that sequester the gonocytes inside of the seminiferous tubules [17]. The tight junctions hinder a direct access to many nutrients and regulatory factors that have to be supplied to the germ cells by the Sertoli cells, constituting the main role of the adult Sertoli cell [17].

Similarly to previously published results, we found that 5hmC is enriched in gene bodies [6, 8, 20, 29, 30]. However, we observed that gain or loss of 5hmC during maturation is associated with different gene regions (Figure 2b and c). Our results indicate a reduced 5hmC enrichment at the TSS, in line with previous findings in adult mouse cerebellum [20], but in contrast to findings in mouse embryonic stem cells, where 5hmC is enriched at the TSS [13].

The median peak length of lost peaks was significantly longer than that of maintained and gained peaks. Hence, the global increase in 5hmC level in Sertoli cells is accompanied by a shift to shorter peaks.

### Functional pathways enriched with 5hmC peaks in juvenile and adult Sertoli cells coincide with cell state function

The pathways enriched with 5hmC peaks that are lost during maturation include morphogenesis, development and cell adhesion, which seem to correlate with the function of the rapidly proliferating Sertoli cells in the young rat. Indeed, these gene ontology categories correspond with those previously found to be upregulated in prepubertal Sertoli cells [21]. Gene pathways pertaining to development or morphogenesis were not enriched in the fraction of genes that gained 5hmC. During the time between 9 and 22 days of age, the expression of tissue development- and neurogenesis-related genes was found to decrease in rat Sertoli cells [21]. The high significance values of tube development, cell localization and projection are particularly interesting in this context, since the Sertoli cell is considered to determine the structure of the emerging seminiferous tubule [31].

Whereas the proliferating prepubertal Sertoli cells seem to lose 5hmC enrichment overwhelmingly in development- and structure-associated genes, the pathways that gain 5hmC during maturation are associated with nucleotide binding, transportation, catabolic and lipid processes, mitochondrion, cytoskeleton and vesicle-mediated transportation. Adult Sertoli cells are known to have a diverse and highly active metabolism with an abundance of mitochondria, and a high expression of metabolism-related genes [31]; this is partly due to the processing of spermatid cytoplasm and the abundance of lipids this entails [32]. It is therefore interesting that multiple pathways associated with metabolism, lipids and mitochondrion were identified as 5hmC-gaining pathways in the adult Sertoli cell. In fact, gene expression data for adult Sertoli cells have shown that the largest proportion of expressed genes was associated with metabolism, with a relatively large number of genes expressed in the transport category; moreover, that Sertoli cells express multiple enzymes involved in general metabolism, lipid metabolism and nucleotide metabolism [16].

Genes related to the cytoskeleton acquire 5hmC peaks during maturation. Cells rely on the cytoskeleton for cell shape and polarity, and for sorting and transporting proteins. However, the terminally differentiated Sertoli cells construct one of the most intricately elaborate cytoskeletons known, which enables these cells to carry out functions that are essential to male fertility, including anchoring germ cells and translocating these toward the lumen of the seminiferous tubule, often through the very cell body of the Sertoli cells [33]. The cyclic process of the spermatogenesis entails a continuous reshaping of the Sertoli cells and requires a dynamic cytoskeleton [33]. Cytoskeleton disruption results in defects in sperm release and cytoplasm phagocytosis [34]. The constant remodeling of the cell shape also requires the presence of proteases, a group of enzymes known to be highly expressed in adult Sertoli cells [31]; genes coding for proteases, however, did not gain 5hmC peaks in our experiments.

Genes coding for lysosome and vesicle-associated pathways gain 5hmC peaks during Sertoli cell maturation. This finding is interesting considering that the Sertoli cell is phagocytotic; the final stage of spermiogenesis involves the removal of spermatid cytoplasm and phagocytosis of residual bodies. These processes as well as the continuous engulfing of apoptotic germ cells lead to the formation of numerous lysosomes and multivesicular bodies in the cytoplasm of Sertoli cells [31].

When using gprofileR to compare the juvenile data set comprising maintained and lost peaks, with the adult data set comprising maintained and gained peaks (Supplementary Figure S4), an overlap in metabolism-related pathways appear. The juvenile animals display 5hmC in metabolism-related genes, but these pathways are not discernible when restricting the analysis to peaks that are lost. Hence, when the maintained peaks are included in the analysis it is evident that juvenile animals also exhibit 5hmC in genes associated with metabolism. Cells at all states possess an inherent metabolism, if 5hmC content is indicative of active transcription it is not surprising that 5hmC is present in genes belonging to metabolism pathways in both immature and mature Sertoli cells. Indeed, the majority of peaks are maintained, reflecting the fact that every cell carries out general physiological metabolism vital for basic cellular functions. The genes necessary for these functions would not be affected by the maturation state of the cell. In additionddition, the adult animals do not display 5hmC enrichment in development-associated pathways. The consistent absence of development pathways in the adult data set confirms this pattern of 5hmC enrichment in active genes.

### Maturational loss of 5hmC in genes that are upregulated in immature Sertoli cells

The number of genes with 5hmC peaks that are gained or lost during maturation would predict that more genes would gain peaks than would lose peaks. In fact, 70% more genes gained than lost 5hmC (Table 1 and Figure 4). However, for the genes that Ryser *et al.* [21] found to be highly upregulated in juvenile Sertoli cells, fewer genes gained (nine genes) than lost (25 genes) 5hmC (Supplementary Table S3). Among the genes that were found to lose 5hmC is *Vasn/ATIA* (loses one out of two peaks); male *ATIA*^*−/−*^ mice have markedly increased apoptosis of the seminiferous tubule and impaired fertility [35]. *NCAM* is another gene that was found to be upregulated in immature Sertoli cells and lost 5hmC peaks during maturation (loses three out of nine peaks); *NCAM* codes for a cell adhesion molecule that has been identified as an important factor in gonocyte–Sertoli cell adhesion in neonatal rats [36]. A third illustrative example is *Col4a1* (loses three out of nine peaks), a component of type IV collagen that constitutes the main component of basement membranes; a decrease in the murine expression of *Col4a1* has been shown to be associated with failure to maintain testicular cord integrity. *Col4a1* is regulated by *Wt1* that shows up in our data set with 2 maintained peaks and 1 that is lost [37].

Analogous to our results, evidence has emerged that 5hmC is enriched in the gene bodies of actively transcribed genes [1, 6, 9, 14, 20, 30]. 5hmC cannot be bound by the transcriptionally repressive methyl-CpG binding domain proteins [38, 39], indicating that presence of 5hmC at the gene body regulates transcription rates by inhibiting the binding of repressive proteins and altering the accessibility of chromatin to the transcription machinery.

It has been suggested that 5hmC influences tissue-specific gene expression during development. Genes that acquired 5hmC between the age of 7 days and adult in mouse cerebellum were involved in cerebellum development and various neuronal functions [8]. Accumulation of 5hmC in the mouse brain during aging has been reported previously with both aging in itself as well as developmental reprogramming as suggested causes [8]. It has also been shown that 5hmC accumulates in the developing mouse cerebellum until adulthood and then becomes stable [20]. However, a recent report demonstrated that 5hmC is reduced in T cells during their lifetime [40]. Future research dedicated to epigenetic modifications in Sertoli cells should broaden the time frame to include more time points in order to further elucidate the possible fluctuations in 5hmC during the entire lifespan of the animal.

All TET proteins are expressed in the juvenile rat testes [41]. Identifying the TET variant that is responsible for the observed increase in 5hmC would give important information about the nature of this maturation.

Our results indicate that a global increase and dynamic redistribution of 5hmC occurs during the maturation of the Sertoli cells. The 5hmC level ceases to increase when Sertoli cells have acquired all the features of the adult cell. Many of the genes expressed in Sertoli cells during testis formation are silenced during spermatogenesis. We show that this change in gene expression is accompanied by a shift in genomic 5hmC distribution, with 5hmC being enriched in actively transcribed genes that define the cell state.

## Materials and Methods

### Animals

Adult male Wistar-Hannover rats were obtained from the section for Comparative Medicine at the Oslo University Hospital, Ullevål. Prepubertal Wistar-Hannover rats were 7-day-old and adult rats were 15-week-old. Animals were purchased from the Møllegaard Breeding and Research Center, Skensved, Denmark, and housed at the Section for Comparative Medicine, Oslo University Hospital, Ullevål. All procedures were approved by the Norwegian Animal Research Authority (Vit 1033), in accordance with the European Convention for the Protection of Vertebrate Animals used for Experimental and other Scientific Purposes (ETS no.123).

### Sertoli cell isolation

Adult Sertoli cells were isolated according to a method described earlier [42] with minor changes (Supplementary Figure S1A). Briefly, testes were excised and placed in 1×HBSS (Hank’s Balanced Salt Solution, cat#14185-052, adjusted to pH 7.4 with sodium bicarbonate, cat#25080-094, Life Technologies, Oslo, Norway). Testes were decapsulated and subjected to enzymatic digestion (collagenase solution (C2674, Sigma-Aldrich, St Louis, MO, USA), trypsin (T5266, Sigma), hyaluronidase (H6254, Sigma), DNaseI (D5025, Sigma)) and washing steps to free the tubular cells by breaking down the interstitial matrix. All enzyme solutions were dissolved in HBSS and the washing steps were carried out in HBSS. After isolation, cells were fixed by suspension in ice-cold 70% ethanol and placed in freezer at −70 °C. Seminiferous tubules from juvenile 7-day-old rats were obtained by testis excision, decapsulation and washing in HBSS. At the age of 6 days, the seminiferous tubule of the rat contains on average 36.0 Sertoli cells and 1.5 spermatogonia [43]. This entails that 96% of the cells in the seminiferous tubule are Sertoli cells at this age. At the age of 9 days, the Sertoli cells constitute 94% of the seminiferous tubule cells [43].

In order to further increase the purity of the isolated Sertoli cells, the samples were labeled for vimentin (Supplementary Figure S1B) and sorted with fluorescence-activated cell sorting (FACS). Ethanol-fixed adult Sertoli cells from the cell isolate described above were transferred to 15 ml tubes and incubation buffer (PBS with 0.1% Tween20 and 0.5% bovine serum albumin) was added. The cells were rinsed twice in incubation buffer and then blocked in the incubation buffer for 10 min at room temperature. Primary antibody was added (vimentin 1:50, Sigma-Aldrich Cat# V6630) and incubated for 1 h at room temperature. After three washes, secondary antibody was added (Alexa Fluor 488 goat anti-mouse 1:500, Invitrogen Cat# A-11029) and the cells incubated for 30 min at room temperature. Cells were rinsed twice and then filtered through a cell strainer cap of polystyrene tubes (75 μm, VWR Cat# 352235). The cells were then sorted using a FacsAria II with the Diva 6 software (BD Biosciences, San Jose, CA, USA, Supplementary Figure S1C). The vimentin-positive and -negative cell fractions were collected during sorting.

### FACS validation

To verify that vimentin-positive cells are Sertoli cells, the sorted vimentin-positive and -negative fractions were stained with SOX9 as an additional Sertoli cell marker [44]. Briefly, 20 000 sorted cells in 200 μl PBS buffer were applied to microscope slides with a cytospin centrifuge (1 000 r.p.m., 5 min). The slides were washed twice with TBST (50 mM Tris, 150 mM NaCl, pH 7.5 with 0.05% Tween 20) and blocked with 5% bovine serum albumin in TBST for 60 min. SOX9 antiserum (Sigma, HPA001758, 1:250) in blocking solution was added and incubated for 60 min at 25 °C. The cells were washed three times for 10 min with TBST and then incubated with the secondary antibody (goat anti-rabbit-Alexa 594, 1:500, Life Technologies) in blocking solution for 60 min at 25 °C. Following two washes with TBST, the slides were incubated for 10 min with 5 μg ml^−1^ 4,6-diamidino-2-phenylindole (DAPI). The samples were then mounted with Mowiol (Calbiochem, Sigma-Aldrich) and dried in the dark for 12 h. Slides were observed with an Axio Observer.Z1 Axio inverted microscope (Carl Zeiss, Oslo, Norway) and quantified using AxioVision Software Release 4.8 (Carl Zeiss). The cell isolate obtained had a purity of at least 95% (Supplementary Figure S1D and E).

### Quantification of DNA modifications by LC-MS/MS

Genomic DNA was enzymatically hydrolyzed to nucleosides essentially as described in [45], added 3 volumes of methanol and centrifuged (16 000 *g*, 30 min, 4 °C). The supernatants were dried and dissolved in 50 μl 5% methanol in water (v/v) for LC-MS/MS analysis of the deoxynucleoside 5hm(dC). A portion of each sample was diluted for the quantification of 5 m(dC) and unmodified deoxynucleosides dA, dC, dG and dT. Chromatographic separation was performed on a Shimadzu Prominence HPLC system (Shimadzu, Kyoto, Japan) with an Ascentis Express C18 150×2.1 mm ID (2.7 μm) column equipped with an Ascentis Express C18 5×2.1 mm ID (2.7 μm) guard column (Sigma-Aldrich). The mobile phase consisted of water and methanol (both added 0.1% formic acid), for 5 m(dC) and 5hm(dC), starting with a 5-min gradient of 5–60% methanol, followed by 6 min re-equilibration with 5% methanol, and for unmodified nucleosides maintained isocratically with 20% methanol. Mass spectrometric detection was performed using an API5000 triple quadrupole (AB Sciex, Oslo, Norway) operating in positive electrospray ionization mode monitoring the mass transitions 258.1/ 142.1 (5hm(dC)), 242.1/ 126.1 (5 m(dC)), 252.1/136.1 (dA), 228.1/112.1 (dC), 268.1/152.1 (dG) and 243.1/127.1 (dT). Statistical analysis of LC-MS/MS data was performed using one-way analysis of variance with Dunnett’s post test, Graphpad Prism version 5.00 (San Diego, CA, USA).

### Immunohistochemistry

Organs were excised and immediately placed in 10% neutral-buffered formalin. After dehydration and paraffin-embedding the organ blocks were cut into 4 μm sections and allowed to dry. The sections were warmed at 60 °C for 1 h, and then deparaffinized in Clear Rite 3 (Thermo Scientific, Waltham, MA, USA) and rehydrated in a series of 100–70% ethanol. Epitope retrieval was carried out in heated Tris-EDTA buffer (10 mM Tris, 5 mM EDTA, pH 9). After incubation in PBST (0.1% Tween20) for 10 min, the sections were blocked (5% bovine serum albumin, 5% goat serum in PBST) for 30 min. Primary antibodies (Vimentin 1:50, Sigma-Aldrich Cat# V6630; 5hmC 1:200, Active motif, La Hulpe, Belgium, Cat# 39769; 5mC 1:200, Diagenode, Liege, Belgium, Cat# MAb-081-100) were added and the sections incubated at 4 °C overnight. After three washes, secondary antibodies were added (Alexa Fluor 488 goat anti-mouse 1:500, Invitrogen Cat# A-11029; Alexa Fluor 594 goat anti-rabbit 1:500, Invitrogen Cat# A-11012) and incubated for 30 min at room temperature. For DNA counterstaining, DAPI (1 μg ml^−1^) was used and sections were covered with mounting medium (Dako, Agilent Technologies, Santa Clara, CA, USA, Cat# S3023).

### Quantification of 5mC-specific and 5hmC-specific fluorescence intensity

Fluorescence images were captured using an Axioplan2 epifluorescence microscope (Carl Zeiss) fitted with fluorescence filters for fluorescein isothiocyanate, TexasRed, and DAPI, and a SPOT RT digital camera (Diagnostic Instruments, Sterling Heights, MI, USA). We tested the linearity of optical density measurements obtained with the instrument using the FocalCheck #1 slide (Life Technologies), which contains fluorescent beads of linearly increasing dye intensity. Expected and measured integrated density of the beads were highly correlated (*R*^2^=0.97). Testicular sections from all developmental stages (days 7, 13, 21 and adult) were mounted on the same microscope slide to allow direct comparison across stages. Slides were stained under standardized conditions (see above). For measurement of nuclear optical density, images of 10–20 high-power fields per stage were captured using strictly identical exposure time for the three fluorescence channels. Exposure times were initially determined for the whole experiment so that no area of the image was overexposed, in order to allow precise quantification of optical density. Monochrome images of red, green and blue channels were captured and imported in ImageJ (National Institutes of Health, Bethesda, MD, USA, version 1.47v). In ImageJ, while viewing nuclei in the DAPI channel, the Cell Magic Wand tool of Theo Walker was used to mark the perimeter of 250–300 nuclei per image. The following cell types were distinguished based on their position in the seminiferous tubuli, nuclear shape and chromatin texture: peritubular cells, Sertoli cells, spermatogonia and spermatocytes. The integrated optical density of the annotated nuclei was measured in ImageJ and further analyzed in R (version 3.1.3).

### 5hmC pull-down

We performed 5hmC pull-down and chemical labeling of genomic DNA using the hMe-SEAL method described by Song *et al.* [20]. An amount of 25 ng of each sample was subjected to Illumina's Nextera Tagmentation reaction at 55 °C for 6 min to simultaneously fragment and add adapters to the DNA. Then, 100 ng of sonicated (~500 bp) unmethylated lambda DNA was added as a carrier (added to the binding buffer to limit/eliminate the possibility of the carrier getting tagmented) during the cleanup with a Zymo Clean & Concentrator Kit (Zymo research, Irvine, CA, USA). 5hmC in the eluted DNA was labeled in a reaction containing 2 μM beta-GT enzyme and 100 μM UDP-N3-Glc at 37 °C for 60 min. The reactions were cleaned again using a Zymo kit and eluted DNA was reacted with 150 μM DBCO-biotin at 37 °C for 90 min. Free biotin reagent was removed by another reaction cleanup using the Zymo kit. Labeled DNA was bound to pre-washed C1 Streptavidin beads for 15 min at room temperature, and then washed six times with the binding buffer (5 mM Tris 7.5, 1 M NaCl, 0.5 mM EDTA, 0.01% Tween-20). Bound (5hmC-containing) DNA was then amplified (without elution) using Nextera index PCR for 13 cycles. Beads were removed by a magnetic rack. The resulting DNA was checked for quality/quantity by Agilent Bioanalyzer and Kapa Library Quantitation qPCR, and then mixed in an equimolar library pool for sequencing on the Illumina HiSeq2500. The input sample is an equal mixture of a small portion of all six samples and removed immediately before binding to the C1 beads. Input was amplified for seven PCR cycles instead of 13.

### Bioinformatic analysis

Duplicated reads were removed from the raw data using PRINSEQ [46]. The remaining reads were aligned with *STAR* version 2.2 ( [ref. 47]) to the *Rattus norvegicus 6* genome downloaded from UCSC and further processed with *samtools* [48] and *bedtools* [49] to produce bam and bed files. To identify enriched regions of 5hmC in adult, 7 days and input data set, we used the peak caller *SICER* v1.1 ( [ref. 50]). *SICER* models the genetic background with random reads based on the input data set, and then identifies clusters of enrichment in a given experimental condition based on this random model. *SICER* was run with window size *w* 200, fragment size 150, gap size *g* 600 and a false discovery rate (FDR) of 0.01. Clusters of enrichment from each of the SICER runs were combined to generate a count matrix by counting reads in each region using the read summarization software *featureCounts* [51]. The count matrix was further processed in *R* and normalization of the data was done with *Voom* [52] transform in the *limma* package [53], which transforms count data to log2-count per million. The data were linear-fitted and differential enriched regions found using eBayes. On the basis of the results, each enriched region was grouped either to belong to the 7 days (lost), adult (gained; FDR<0.05) or common (maintained) group (FDR>0.05). The perl script *annotatePeaks* in *HOMER* [54] was used to annotate the 5hmC-enriched regions with various gene features like nearest gene, distance to TTS, gene region (5′UTR, promoter, exon, intron, intergenic, 3′UTR, non-coding) and so on. The annotated regions were merged with values from the statistical analysis and the final data set was ready to be analyzed.

The gene ontology database DAVID was used for pathway analysis (search performed on 3 June 2016; https://david.ncifcrf.gov [55]), as well as Metascape (search performed on 3 June 2016; http://metascape.org [56]).

Enrichment analysis was performed with gProfileR, an R-package derived from the web-based gProfile toolset [57]. gProfileR was run with a FDR threshold of 0.05. The enrichment plot was made using the ggplot2 package in R. We divided each region based on its enrichment into two groups; *Juvenile 5hmC* or *Adult 5hmC*. The category *Juvenile 5hmC* includes genes with peaks that are lost and maintained during maturation. The category *Adult 5hmC* includes genes with peaks that are maintained and gained during maturation.

## Figures and Tables

**Table 1:** Sequencing and mapping results

	*Peaks*	*Intragenic peaks*	*Intragenic peaks (%)*	*Genes*
Lost	9 465	4 258	45	1 927
Gained	10 116	5 275	52	3 284
Maintained	78 627	34 337	44	9 559
Total	98 208	43 870	45	10 725

Peaks that only appear in the juvenile animals and are lost during maturation are designated Lost; peaks that are gained during maturation and appear only in the adult animals are designated Gained; and peaks that appear in both ages of animals are designated Maintained. Number of identified peaks, number and percentage of intragenic peaks, number of genes that contain the peaks.
