# Supplementary material for: Genome-wide profiling of DNA 5-hydroxymethylcytosine during rat Sertoli cell maturation
Source: Cell Discov. 2017 May 9;3:17013–. doi: 10.1038/celldisc.2017.13 (PMC5423031; doi:10.1038/celldisc.2017.13)
Supplement: Supplementary Figures [file celldisc201713-s1.pdf]

Supplementary Figure 1

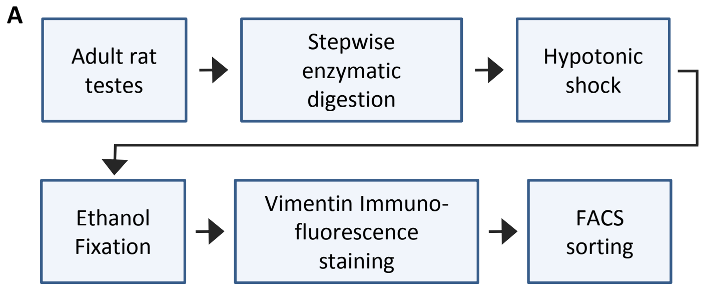

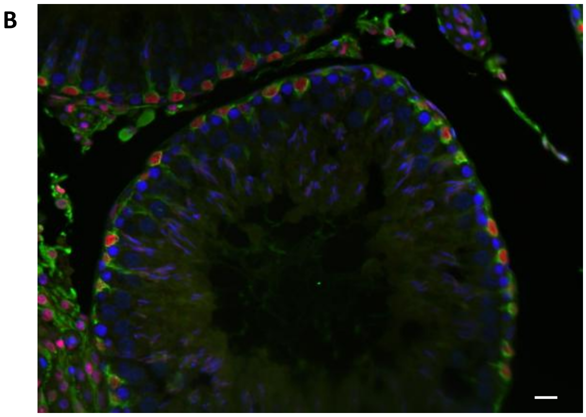

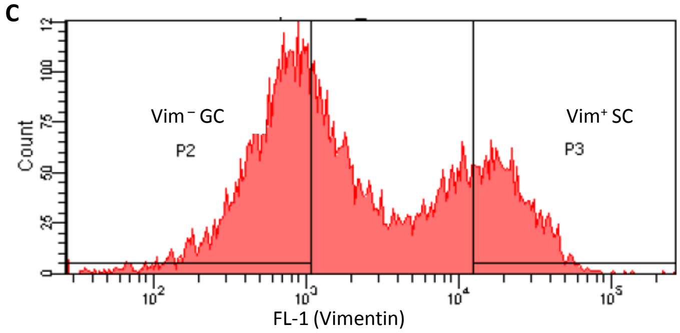

D

Percentage of positive cells

Sorted cells	Vimentin	SOX9
Vim⁺ SC	95.8%	96.6%
Vim⁻ GC	0.8%	0.8%

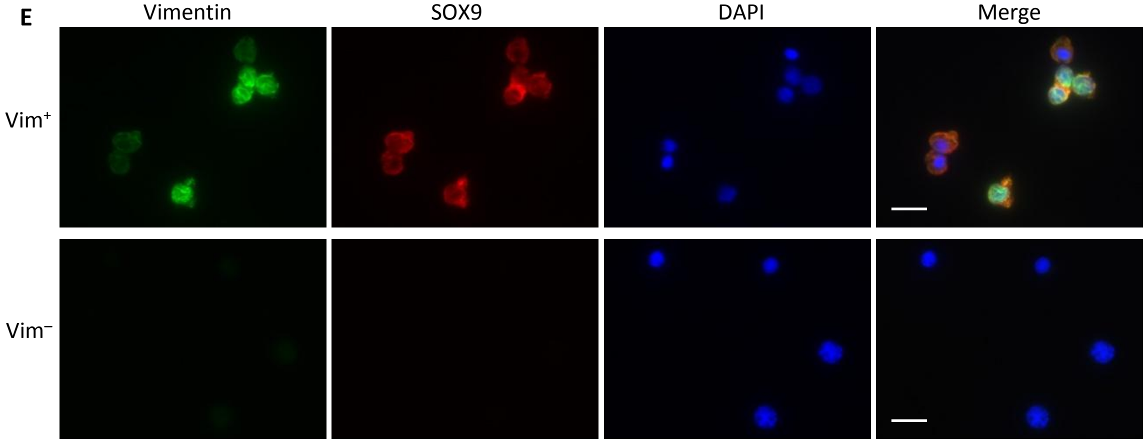

Supplementary Figure 2

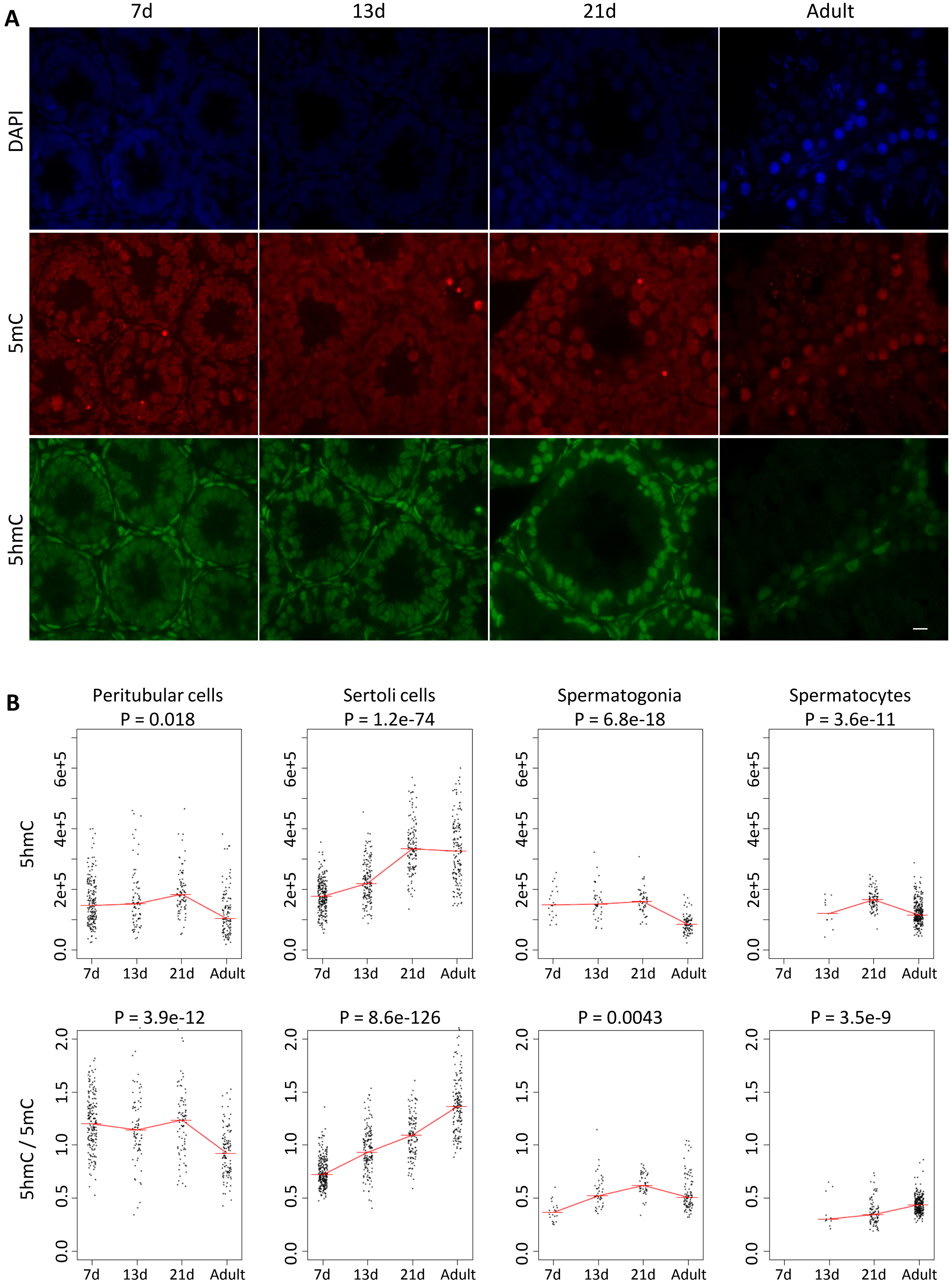

Supplementary Figure 3

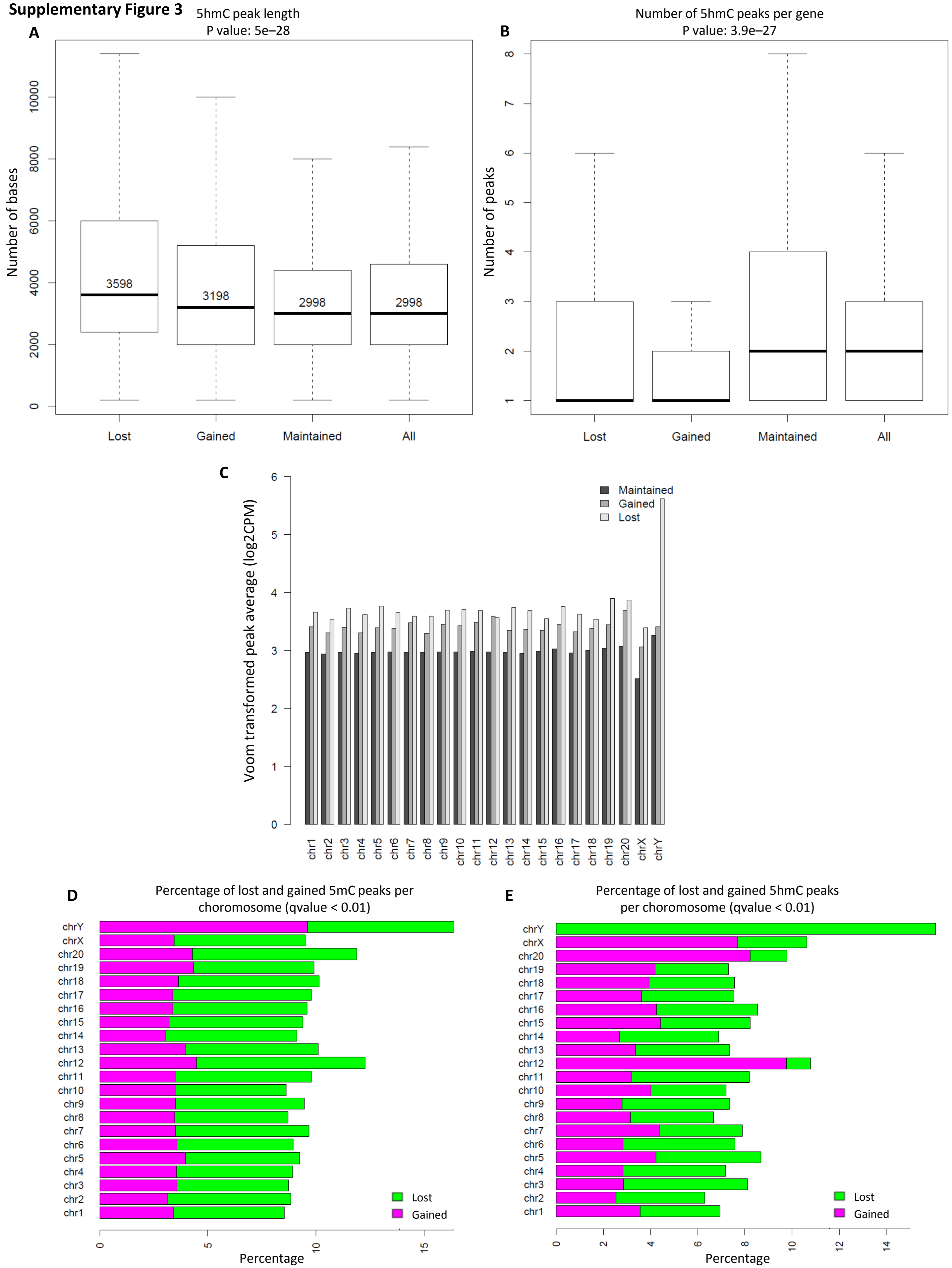

Supplementary Figure 4

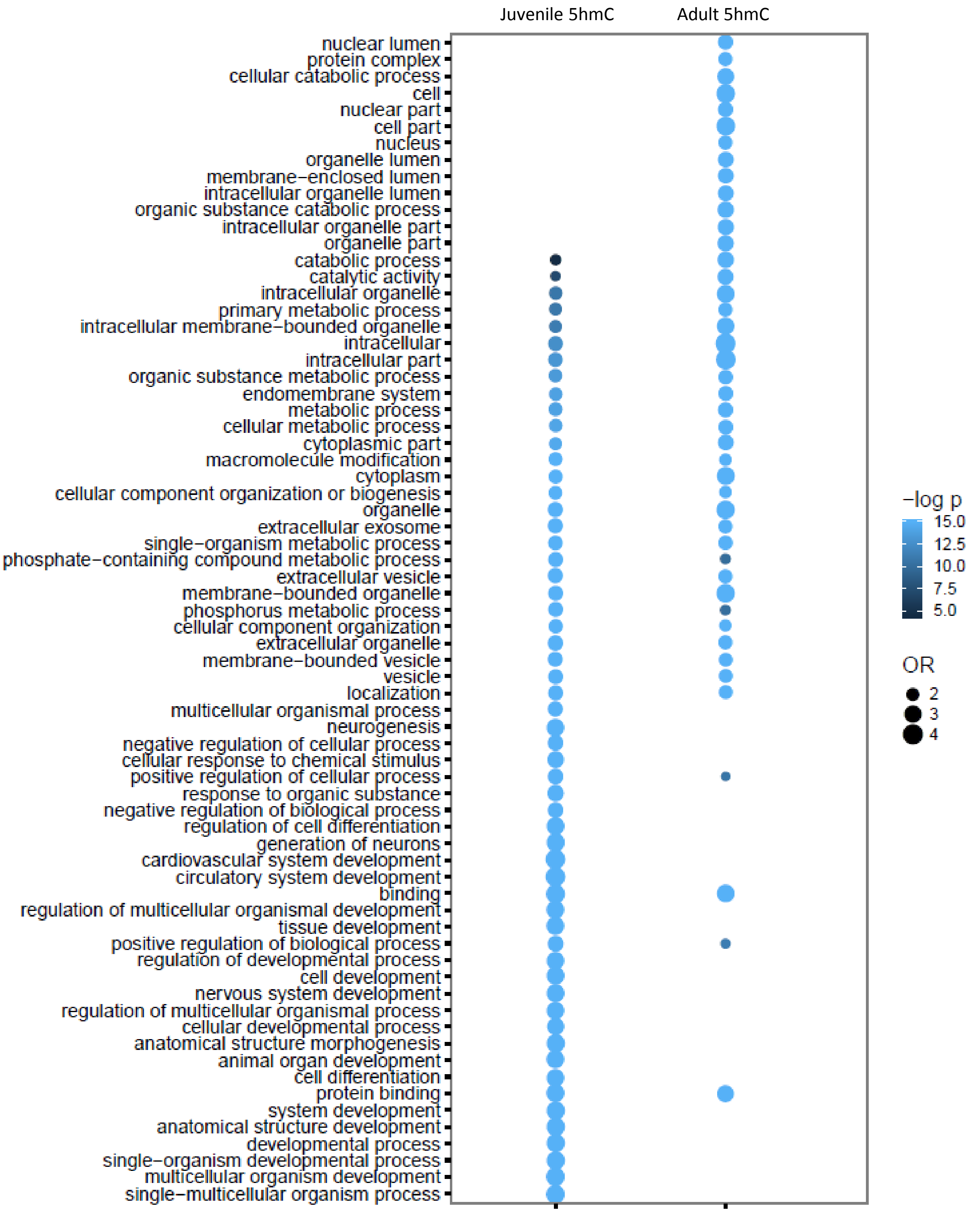

Supplementary Figure 1. Isolation of adult Sertoli cells and validation of sorted cell fraction purity. A) Schematic overview of the isolation of adult Sertoli cells from whole testis. B) Vimentin stained cells in section of adult rat testis, red 5-hmC; green vimentin; blue DAPI. C) FACS sorting of cells with sorting gates, vimentin negative germ cell fraction (Vim^{-} GC) and vimentin positive Sertoli cell fraction (Vim^{+} SC) D) Percentage of sorted cell fractions that are vimentin negative and positive, and SOX9 negative and positive, respectively. E) Immunofluorescence staining of sorted cell fractions, green vimentin; red SOX9; blue DAPI, scale bars 20 μm.

Supplementary Figure 2. Quantification of 5hmC and 5hmC/5mC. A) Immunofluorescence images and B) quantification of 5hmC and 5hmC/5mC, at ages 7 days, 13 days, 21 days, and 15 weeks (adult). Signal intensity measured relative to DAPI intensity (arbitrary units); n=250-300 per image. Scale bar is 10μm.

Supplementary Figure 3. A) 5hmC peak length and B) Number of 5hmC peaks per gene for lost, gained, maintained, and all peaks, Student's t-test C) Voom transformed peak averages, mean of all log2CPM peak intensities per chromosome D) Percentage of gained and lost 5mC peaks per chromosome E) Percentage of gained and lost 5hmC peaks per chromosome

Supplementary Figure 4. Visualization of 5hmC-enriched pathways using gprofileR. Category Juvenile 5hmC comprises all genes that include peaks that are maintained or

lost during maturation; category Adult 5hmC comprise all genes that include peaks that are maintained or gained during maturation. All genes that contain a 5hmC peak at any time point were sorted exclusively on the basis of losing or gaining a peak, the resulting categories are designated One lost peak and One gained peak, respectively.
