# Supplementary material for: Genome-wide profiling of DNA 5-hydroxymethylcytosine during rat Sertoli cell maturation
Source: Cell Discov. 2017 May 9;3:17013–. doi: 10.1038/celldisc.2017.13 (PMC5423031; doi:10.1038/celldisc.2017.13)
Supplement: Supplementary Tables [file celldisc201713-s2.pdf]

Supplementary Table 1. Input reads, filtered reads, uniquely mapped reads and multiply mapped reads resulting from the sequencing and mapping of Sertoli cell DNA from three juvenile (7 day-old), and three adult rats.

File	Input reads	Filtered reads	Uniquely mapped reads	Number of reads mapped to multiple loci k10
**7d1 (7 days)**	17198802	16073453	12130622	836758
**7d2 (7 days)**	26457503	24281928	17265583	1475578
**7d3 (7 days)**	52508307	44467712	29561807	2360617
**Input**	18528781	17303914	11570289	1161612
**SC1 (Adult)**	37399781	32997766	23867012	1898692
**SC2 (Adult)**	24981216	22708916	16887801	1238683
**SC3 (Adult)**	6587944	6280824	4461372	363257

Supplementary Table 2. Gene pathways that are enriched with 5hmC that is lost and gained during maturation of Sertoli cells. Ten of the top twenty pathways are selected for representation (bold)

Lost			Gained		
	**Pathway**	**Enrichment score**		**Pathway**	**Enrichment score**
1	**Cell morphogenesis**	**12.8**	1	**Nucleotide binding**	**10.0**
2	**Cell adhesion**	**11.2**	2	Ion binding	8.8
3	**Cell projection**	**10.1**	3	**Intracellular protein transport**	**7.2**
4	Membrane fraction	8.4	4	**Catabolic process**	**6.7**
5	Blood vessel development	7.6	5	**Membrane-enclosed / intracellular lumen**	**6.6**
6	Actin cytoskeleton	6.7	6	**Mitochondrion**	**5.9**
7	**Kinase activity**	**6.4**	7	**Cytoskeleton**	**5.5**
8	Cytoskeleton organization	6.0	8	Golgi apparatus	5.4
9	**Extracellular matrix / Basement membrane**	**6.0**	9	**Lysosome**	**4.8**
10	Enzyme binding	5.9	10	Membrane/Vesicular fraction	4.4
11	Ion binding	5.9	11	**Vesicle-mediated transport / Endocytosis**	**3.9**
12	**Organ development**	**5.8**	12	Protein dimerization activity	3.8
13	**Cell junction**	**5.7**	13	Organelle membrane	3.5
14	**Cell localization**	**5.6**	14	Peroxisome	3.3
15	Fibronectin	5.5	15	Chromosome organization	3.2
16	**Tube morphogenesis**	**5.5**	16	Carbohydrate catabolic process	3.2
17	Actomyosin	5.4	17	**Cytoplasmic vesicle**	**3.2**
18	Extracellular matrix	5.4	18	DNA metabolic process	3.2
19	**Cytoskeleton**	**5.1**	19	Vesicle coat	3.0
20	Regulation of cell adhesion	4.7	20	**GTPase regulator activity**	**2.8**

Supplementary Table 3. The most upregulated genes in immature Sertoli cells according to Ryser et al.

"Gene expression profiling of rat spermatogonia and Sertoli cells reveals signaling pathways from stem cells to niche and testicular cancer cells to surrounding stroma." BMC Genomics 12: 29. (2011)

	Number of peaks		
**All upregulated genes**	**Lost**	**Maintained**	**Gained**
Asam	0	6	0
Boc	0	0	0
Cdh11	11	5	0
Cdh22	0	2	0
Col4a1	3	6	0
Itgav	0	0	0
Mdk	0	2	1
Pdgfc	1	2	0
Pvr	0	1	0
Tpbg	0	1	0
Vasn	1	1	0
Col4a2	0	0	0
Cthrc1	0	1	0
Cxcl13	0	2	0
Fbn1	12	4	0
Gpc1	3	6	0
Madcam1	0	0	0
Ncam1	3	6	0
Npy	0	2	0
Pap	1	12	0
Pap3	0	0	0
Sfrp1	1	3	0
Spp1	0	4	1
Thbd	1	0	0
Torid	0	0	0
Dbn1	0	2	0
Mybpc3	0	3	0
Nes	0	1	0
Pdlim2	0	3	0
Pdlim3	5	6	0
Tmod1	2	7	1
Trim2	0	10	0
Tubb2b	0	1	0
Cdkn2a	0	0	0
Cdkn2b	0	0	0
Mtsg1	0	0	0
Ech1	0	0	0
Tmem98	0	3	0
Ddah2	1	1	0
Cbr3	0	1	0
Cox6a2	0	1	0
Cp	0	1	0
Crym	0	1	0
Dio3	0	0	0
Gpx7	0	0	0
Gsta2	0	1	0
Idh2	0	1	0
Mgst2	1	28	0
Pygl	0	3	0
SelM	0	2	0
Hspb1	0	3	1
Gpr83	0	3	1
Kdr	1	8	0
Ntrk1	0	1	0
Scn3b	1	6	0
Scn4b	1	2	0
Slc15a2	1	2	1
Slc26a3	0	1	0
Smstr28	0	0	1
Sntg2	0	4	0
Tacstd2	0	0	0
Trfr2	0	0	0
Arhgef4	0	2	0
Cpne8	0	12	0
Ptpns1	0	4	0
Ltbp2	0	6	0
Rerg	0	1	0
Rrad	0	1	0
S100a3	0	1	0
S100a5	0	0	0
Smoc2	2	10	0
Giot1	0	2	1
Lmcd1	0	4	1
Nr4a1	0	1	0
Nupr1	0	3	1
Rgc32	0	1	0
Sox18	0	0	0
Tead2	0	1	0
Znf292	3	6	0
Znf704	0	0	0
Bmf	0	2	0
Inhba	1	2	0
Mgp	0	1	0
Apoe	0	2	0
Dscr1l1	0	7	0
Efna1	0	1	0
Gap43	5	17	0
Ntf5	0	1	0
Penk-rs	0	0	0
Plxnb1	0	0	0
Pnoc	1	0	0
Rogdi	0	0	0
Adam10	1	1	0
Adam33	1	1	0
Htra3	1	4	0
Masp1	1	6	0
Plau	0	1	1
Plat	0	2	0
Plxnc1	0	0	0
Tessp6	0	1	0
Ube2c	0	2	0
**Average**	**0.7**	**2.8**	**0.1**
**Median**	**0**	**1**	**0**

**Number of genes**	**101**

	Number of peaks			
**Genes with lost peaks**	**Lost**	**Maintained**	**Gained**	**Sum**
Cdh11	11	5	0	16
Col4a1	3	6	0	9
Pdgfc	1	2	0	3
Vasn	1	1	0	2
Fbn1	12	4	0	16
Gpc1	3	6	0	9
Ncam1	3	6	0	9
Pap	1	12	0	13
Sfrp1	1	3	0	4
Thbd	1	0	0	1
Pdlim3	5	6	0	11
Ddah2	1	1	0	2
Mgst2	1	28	0	29
Kdr	1	8	0	9
Scn3b	1	6	0	7
Scn4b	1	2	0	3
Smoc2	2	10	0	12
Znf292	3	6	0	9
Inhba	1	2	0	3
Gap43	5	17	0	22
Pnoc	1	0	0	1
Adam10	1	1	0	2
Adam33	1	1	0	2
Htra3	1	4	0	5
Masp1	1	6	0	7
**Average**	**2.5**	**5.7**	**0**	**8.2**
**Median**	**1**	**5**	**0**	**7**

**Number of genes**	**25**

	Number of peaks			
**Genes with gained peaks**	**Lost**	**Maintained**	**Gained**	**Sum**
Mdk	0	2	1	3
Spp1	0	4	1	5
Hspb1	0	3	1	4
Gpr83	0	3	1	4
Smstr28	0	0	1	1
Giot1	0	2	1	3
Lmcd1	0	4	1	5
Nupr1	0	3	1	4
Plau	0	1	1	2
**Average**	**0**	**2.4**	**1**	**3.4**
**Median**	**0**	**3**	**1**	**4**

**Number of genes**	**9**

**Genes with lost and gained peaks**	**Lost**	**Maintained**	**Gained**	**Sum**
Tmod1	2	7	1	10
Slc15a2	1	2	1	4
**Average**	**2**	**5**	**1**	**7**
**Median**	**2**	**5**	**1**	**7**

**Number of genes**	**2**
